# Navigation of Ships in Channel Bends under Special Conditions Using Sensors Systems

**DOI:** 10.3390/s22228783

**Published:** 2022-11-14

**Authors:** Vytautas Paulauskas, Ludmiła Filina-Dawidowicz, Donatas Paulauskas

**Affiliations:** 1 Marine Engineering Department, Klaipeda University, H. Manto Str. 84, LT-92294 Klaipeda, Lithuania; 2 Faculty of Maritime Technology and Transport, West Pomeranian University of Technology, Ave. Piastów 41, 71-065 Szczecin, Poland

**Keywords:** maritime transport, navigational channel, channel bend, navigational safety, sensors for navigation

## Abstract

Navigational channels and approaches to ports may have bends that constitute the specific sailing conditions for ships. A vessel’s entrance into a bend and its safe passing depends on the ship’s position accuracy, turn angle, and internal and external forces influencing the ships, as well as the captain’s or pilot’s experience. In order to assure a ship’s safe navigation under specific conditions, the possibility to measure individual ship movement parameters with the use of special sensors is needed to accurately calculate the ship’s trajectory considering the specific dimensions of ships. Moreover, hydro-meteorological and hydrological limitations for ships with different parameters and maneuverability should be evaluated in advance. The article aims to develop the methodology for calculating ships’ route trajectory in channel bends and approaches to ports under special navigational conditions. The mathematical model that may be used to calculate wind velocity limitations and distance crossed by a ship during maneuvers, depending on the ship’s maneuverability, hydro-metrological, and hydrological conditions, was elaborated. The methodology was verified by the example of a few ships entering specific channel bends. Wind velocity limitations depending on wind direction for the SUEZMAX tanker and other selected types of ships during crossing navigational channel bend near Klaipeda port were calculated. The presented theoretical basis may be used by ships’ captains and pilots who plan and perform operations of vessels’ crossing the approaches to ports and navigational channel bends, as well as by navigational channels designers who plan the channel’s parameters in difficult geographical and navigational conditions. Its application may influence the safety increase of maritime transport in limited or specific areas.

## 1. Introduction

Bends may constitute the sections outside and/or inside a port’s navigational channels and approaches to harbors [1,2]. Their location may be influenced by geographic configuration, infrastructural limitations, set shipping routes, etc. The width of the ship’s sailing pass within the channel bends is greater than the pass width in straight channels sections, because during the ship’s turning the growth of drift angle, a decrease of the ship’s speed, and an increase of wind and current impact the ship [3,4]. At the same time, the external forces, which are created, among other things, by wind, current, waves, and shallow water, affect the increase of drift angle and the ship’s sailing pass [5,6]. Therefore, the accuracy of the ship’s position determination at the entrance to the channel bends is important to set the requested width of navigational channel bends and the whole channel to ensure safe navigation for different types of vessels [3,7,8,9,10].

Moreover, while planning and performing ships’ sailing within channel bends, it is necessary to take into account the impact of human factors. The ship’s captain and/or pilot knowledge, quality of conducted measurements of the ship’s position and movement parameters, as well as local traditions, habits and rules, may influence the ship’s trajectory during passing the bend [11,12].

Analysis of accidents that occurred during big ships crossing the channel’s bends in different navigational channels shows that in many cases, ships were not prepared in advance to make safe maneuvers in specific and complicated conditions due to the insufficient qualification of ships captains and port pilots [12,13]. In some situations, ship captains and pilots did not consider the ships’ technical parameters that influence ships’ behavior afloat and made direct mistakes while crossing navigational channel bends [14]. It should be noted that professional education of staff supported by advanced knowledge of ship maneuverability and navigational conditions can increase the safety level of ships crossing navigational channels bends in special navigational or/and hydro-meteorological conditions with external assistance (e.g., tugs) or without it [15,16].

**Figure 1:**
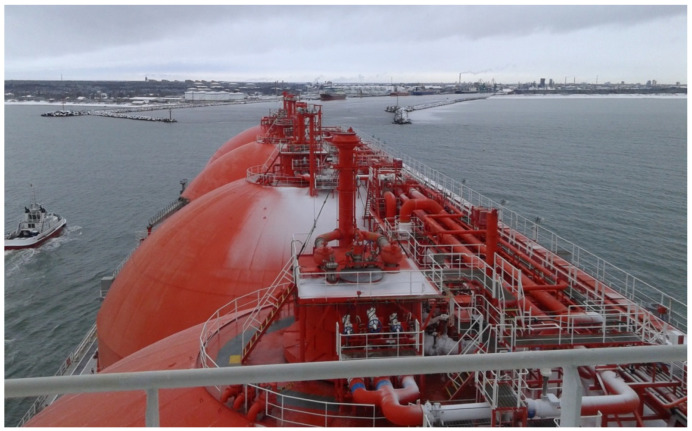
An LNG tanker crossing the approach channel bend close to Klaipeda port gate (own picture).

In some cases, the bends of approaches to ports and port navigational channels are located close to port gates (breakwaters), and the additional challenges for the ship’s safe navigation appear and are related to difficult hydro-meteorological conditions and the lack of accurate information on vessel’s movement parameters (Figure 1) [2,17]. During maneuver planning before crossing a navigational channel bend, it is important to evaluate the possible ship’s trajectory considering different factors’ influence on the ship. In some channel bends, the ships’ engine reverses may be needed to enable vessels to cross safely navigational channel bends [18].

The problem becomes more crucial when bigger and bigger ships have to cross the existing channel bends. On the one hand, in order to ensure the accepted level of the ship’s navigation safety, investments in infrastructure may be made to deepen channels. These investments allow to create more space for the ship’s safe maneuvering; however, this solution is time-consuming and very cost intensive. On the other hand, more accurate planning of the ship’s trajectory while passing the channel bends may allow ships to cross the channels safely. In this case, the trajectory should be planned considering the detailed impact of external forces on the ship, as well as the results of accurate measurement of these forces and ship motion parameters in the current time of ship’s movement. This planning may also be supported by data provided from sensors systems, e.g., real-time kinematics positioning (RTK) that is a technology of precise measurements using satellite navigation [19].

The conducted literature review revealed that the problem of the ship’s trajectory planning during crossing navigational channels bends under special conditions was not analyzed in detail. Therefore, an appropriate theoretical basis and its practical implementation allows calculating in advance the ship’s sailing parameters and trajectory needed to cross the channel bends in limited areas can be beneficial and may impact the increase of maritime transport safety.

The article aims to develop the methodology for calculating ships’ route trajectory in navigational channel’s bends and approaches to ports under specific conditions and using sensor systems. The proposed approach is designed to increase the precision in determining the planned trajectory of vessel traffic.

The article includes a literature review Section, describing current research results in the subject area analyzed. The Methodology Section shows the way chosen to conduct the research, as well asthe mathematical model used to conduct calculations. The outcomes of application of proposed methodology are presented in Results Section, where computational and experimental results were investigated. In order to sum up, the discussions and conclusions are drawn.

## 2. Literature Review

Navigational channel bends located near ports may have different designs [20], including radius of curvature, width, location of transition zones from the straight channel section to the widened bend, etc. It should be mentioned that in some ports, there are bends with very sharp or space-consuming specific turns within approaches and inside navigational channels, as it occurs in ports of Landskrona (Sweden), Stockholm (Sweden), Southampton (United Kingdom), Klaipeda (Lithuania), and other harbors [18,21,22]. During a channel’s design, the possible maximum parameters of the ship that allows their safe maneuverability during crossing channel bends in a limited area, should be considered. It is crucial to minimize the ship’s engine maneuvers while crossing these sections. However, it may be very problematic to implement in archipelagos, fiords, and other similar areas [21,23,24]. 

**Figure 2:**
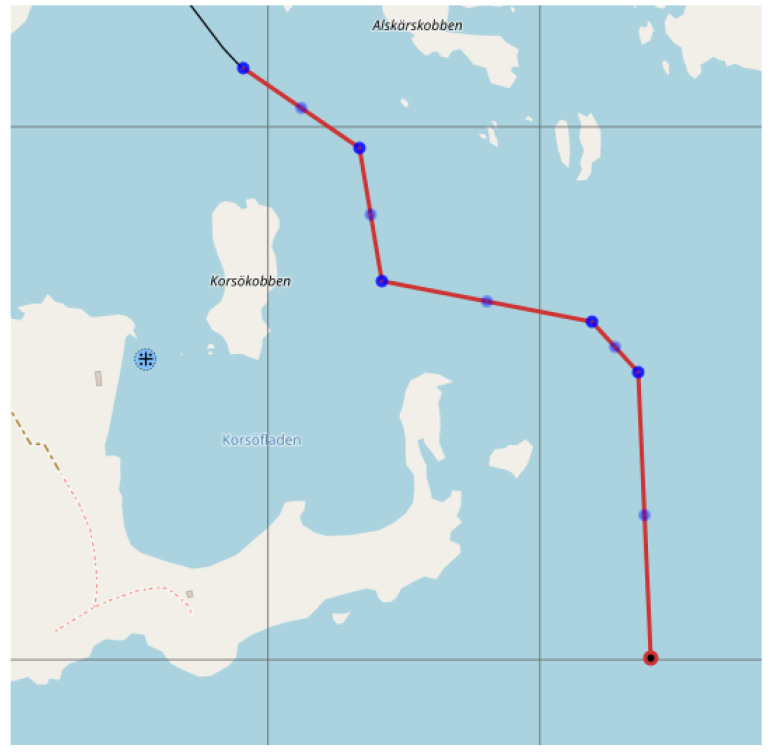
The navigational way near Korsakobben Island (Stockholm archipelago) with bends in the channel [21].

As an example, the navigational channel to/from Stockholm port near Korsakobben Island (Stockholm archipelago) in Sweden can be presented (Figure 2) [21]. Furthermore, due to the global trend of ship dimensions increasing and ports thus taking efforts to attract big ships for cargo handling and other services within existing channels’, the navigational conditions become more challenging.

**Figure 3:**
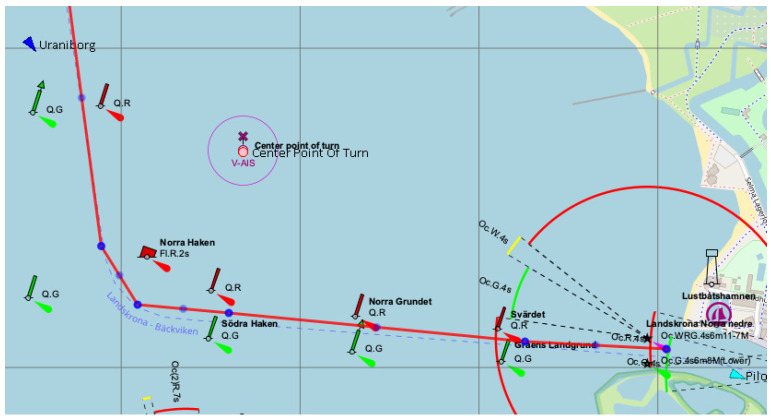
Approach channel bend near Landskruna port, where: QG, QR, “Nora Haken”, etc.—names of buoys; Oc.WRG.4s6m11-7M – characteristics and visibility distance of the lighthouse [21].

For example, some ports in Scandinavia have very specific designs for their approaches. While crossing navigational channel bends in these areas, ships should adjust its maneuvers to specific conditions [21]. Such conditions occur near Landskruna port in Sweden, where the approach channel bend has a turn angle of about 85 degrees (Figure 3).

**Figure 4:**
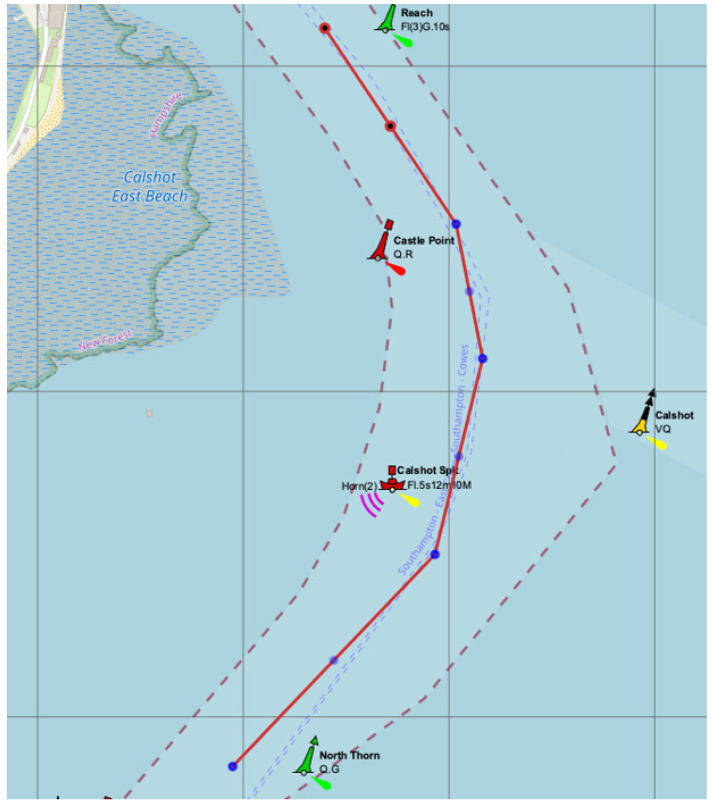
Approach channel bend near Southampton port, where: QG, VQ, QR, “Cals hot”, etc. – names of buoys; Horn(2)Fl.5s12m10M – characteristics and visibility distance of the lighthouse [21].

Another example of a seaport with a complex design of approach channel is Southampton port located on the South Coast of the United Kingdom, where the navigational channel has a bend with turn angle about 90 degrees (Figure 4) [21].

**Figure 5:**
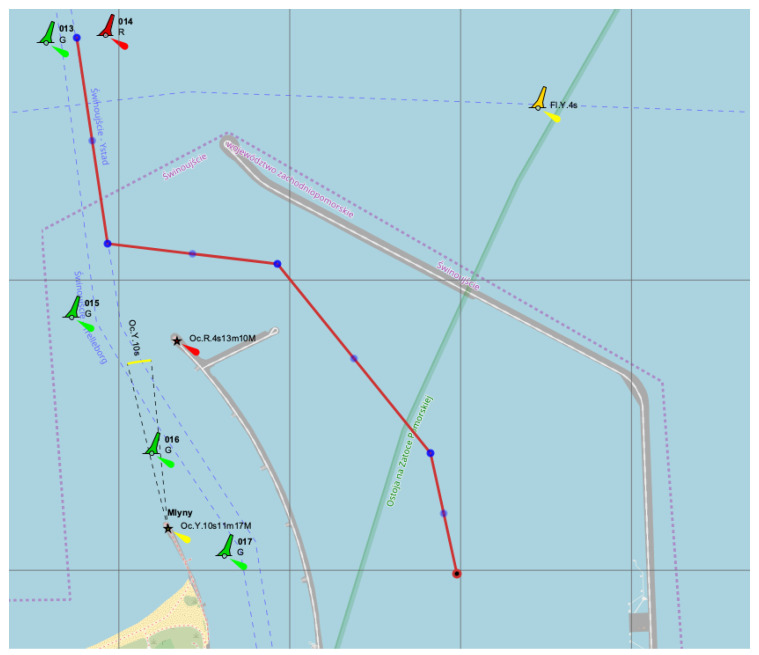
Navigational channel bend of Świnoujście port to LNG terminal area and ship’s sailing way, where: 013G, 014R, etc. – names of the buoys, Oc.Y.10s11m17M, Oc.Y.10s11m17M – characteristics and visibility distance of the lighthouses [21].

The approach to Świnoujście port in Poland and fairway connecting ports in Świnoujście and Szczecin also have unique shapes (Figure 5). Due to the needed turns and specific navigational conditions within the fairway, big ships must use tugs and/or pilot services to safely enter ports.

**Figure 6:**
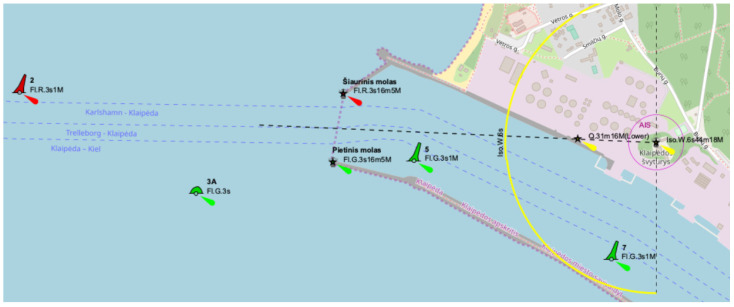
Navigational channel bend close to the gate of Klaipeda port, where: 2 Fl.R.3s1M, 3A Fl.G.3s, etc.—names of buoys; Iso.W.6s44m18M – characteristics and visibility distance of the lighthouse [21].

The navigational channel bend near Klaipeda port has a turn angle of about 25 degrees (Figure 6). It should be highlighted that this channel bend is located close to the oil terminal quay walls, where waves and strong currents appear that penetrate from the open sea. Additionally, a very often strong wind flow with a perpendicular direction to the channel is observed and influences the maneuvering operation’s safety [25].

Within the shape of navigational channels in the sections located after the bends, an additional channel width is required because the ship’s position after crossing the bend may fluctuate [26]. Therefore, the methodology allowing to calculate the ships trajectory considering the required width of channels bends could be very useful for the channels’ designers to take decisions related to optimization of investments, volume in navigational channels building, or modernization, as well as ensuring navigational safety during the ships sailing under specific conditions and within a limited area.

Existing PIANC (Permanent International Association of Navigational Congress) harbor approach channels design guidelines [23], national recommendations for maritime works (e.g., Spanish [7]), and other outlines could be used for the navigational channel’s design. However, significant fluctuation of proposed approaches and achieved results is observed [1,9,14,27] that makes it difficult to apply them for channel bend optimization in specific conditions.

The conducted available literature analysis revealed that theoretical studies on ship’s maneuvers in the ports channels and waterways were conducted and addressed ships crossing the channel bends [2,3,28]. Attention was paid to the necessity to conduct a risk assessment of ships’ maneuvering based on simulation data [29]. Moreover, ships’ trajectory planning algorithms using navigational data were proposed [30]. However, mentioned studies mainly consider the ships’ direct turnings in channel bends without using additional reverses by the ships’ engines.

The available studies highlight the need to increase maritime transport safety [31,32], avoiding ships collisions, and groundings occurring in ports’ areas, as well as in channel bends [13,17,33]. The methods to avoid ship collisions and grounding were analyzed in detail [16,34]. These publications take into account the ships’ direct maneuvers without additional maneuvers, or using additional assistance by vessels (e.g., tugs).

Considering the hydrodynamic effect between ships passing and mooring to quay walls, navigational channels located close to moored ships were investigated [9,17]. The specific behavior of ships was analyzed considering their size, the maritime conditions, and mooring location [35]. The model that took into account ship maneuvering dynamics and associated hydrodynamic actions emerging from different rudder angles and forward speed effects was proposed [36].

Several research studies present the results of simulations conducted for restricted port regions and channels to optimize port water areas and channel parameters [29,37,38,39,40]. Mentioned studies have paid attention mainly to the effectiveness and assessment of the existing navigational situations for the different ship types and sailing conditions. However, situations when there is a necessity to perform a large number of the ship’s engine maneuvers, while crossing very restricted water areas or channel bends, were not described in detail.

The current studies also focus on the investigation of ships’ trajectories and maneuvers in restricted water areas using tugs or other types of assistance [15,41,42,43], as well as hydrodynamic and other effects between passing ships and moored ships, sailing ships and banks [9,44,45]. It is emphasized that different conditions influence the ships’ maneuvers that include i.a. wind and waves [46], shallow waters [47], current, etc. It was shown that weather conditions vary significantly with regard to the maneuvering area [10]. Despite the detailed examination of ships maneuvering in restricted areas, available studies do not consider real complex conditions influencing the ships’ maneuvers while passing the channel bends.

New technology usage, such as the Automatic Identification System (AIS), RTK and other innovative equipment, is very substantial to increase navigational safety in restricted port areas and channel bends [4,48,49]. To support quantitative safe ship operation guidelines, ship trajectory data based on AIS information may be applied [50]. These data may be used to improve the reliability of ships’ position information, especially in ports and other safety-critical areas [51]. The application of land-borne laser rangefinder measurements for navigation safety assessment was proposed [52]. The quantification methodology to assess potential risk among vessels ahead of a collision situation was introduced to support e-navigation [53]. The ship’s maneuvering area and collision probability were calculated considering uncertainty areas of the ship’s plan geometry at a given probability level for typical equipment applied in existing pilot systems [54]. The algorithm designating direct hazards for the ship in an encounter situation for numerous ships was elaborated [55]. The vessel trajectory data were collected [56], and methods to analyze these data were proposed, considering information services modules, navigational assistance services, and traffic organization services [57]. Additionally, vessel traffic services (VTS) are contributing to the safety and efficiency of maritime traffic [58]. These systems support keeping track of ships’ movements in limited geographical areas using radars, radiotelephony, closed-circuit television, and other equipment [59]. Moreover, the approach feasible to predict maneuvering trajectories of existing or new-build vessels and to estimate the evasive velocity in the way of contact before grounding was presented [60]. The results of these studies may support the navigation process planning; however, this process should additionally consider real external factors impacting ships that may be calculated in advance.

Subject literature includes research studies on ships trajectory planning and related risk factors [61], their frequency and the relationship between risk factors and accidents during ships sailing in complicated and specific navigational conditions [3,14,19,40,62]. Crew incompetence due to their poor education is observed in more than 75–80% of accidents and incidents in navigational channels [11,12,13]. In some cases, a ship’s crew may have very limited knowledge about the maneuverability of big sea-going vessels and real trajectories. Moreover, the lack or insufficient planning process made for ship’s crossing channel bends may lead to up to 80% of ship accidents and incidents due to mistakes made in the preparation of such processes [63]. Not enough practice on the part of a ship’s crew on sea-going ships and insufficient knowledge about the maneuverability of big sea-going vessels are observed in more than 40% of accidents and incidents occurring during towage operations [13]. It should also be noted that 20% of accidents and incidents during the channel’s bends crossing process took place due to inappropriate weather conditions, poor visibility, and the unavailability of accurate environmental and vessel movement data in real-time [64].

The information mentioned above underlines the fact that ships sailing through channel bends require more preoperational work made by the vessels’ masters, especially if there is no possibility of using tugs. Ships sailing in such specific conditions often deal with the increased probability of emergency situations occurring when it is necessary to make a rational decision very quickly without the possibility of additional preparation. Therefore, ships’ sailing trajectory calculation in advance is crucial for the safe crossing of sharp and extensive bends within channels.

The results of the conducted literature review confirm the need to develop a methodology that may be used to calculate the ships’ route trajectory in the navigational channel’s bends and approaches to ports under specific conditions.

## 3. Methodology

### 3.1. Steps of Research Methodology

**Figure 7:**
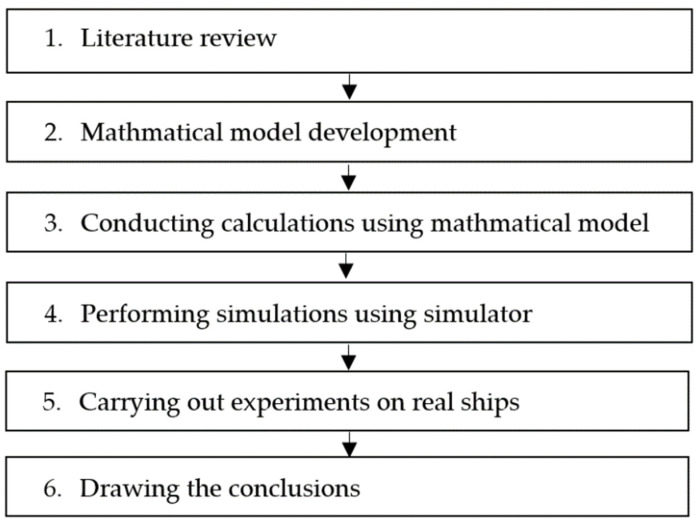
The steps of research methodology.

The following steps of the research methodology were used to conduct the research (Figure 7). After conducting the literature review, the mathematical model was developed.

Based on the presented methodology, the theoretical calculations of possibilities for the ships to cross channel bends in special conditions carrying out a minimum number of requested ship’s maneuvers were performed. For the calculation of the real ship’s trajectory in channel bends, the maximal distribution method was used utilizing data achieved by conducting experiments on simulators and real ships. The maximal distribution method could be applied in the case at least five measurements were carried out.

In order to verify theoretical calculations and the practical application of the presented methodology, experiments were performed with the help of a simulator and on real ships. Simulations were carried out using a full mission simulator “SimFlex Navigator” (Force Technology product), which analyzed similar maneuvers of real ships, considering the set forces acting on the ship crossing the channel bends. Experiments performed on real ships covered two limited areas with specific navigational conditions.

Then, the results were analyzed, discussions and conclusions were drawn, and the directions of future research were outlined.

### 3.2. Mathematical Model

For ships crossing the channel bends, it is necessary to accurately calculate the additional forces and moments required to compensate for external forces [65]. Besides, additional power and output of the compensated impact of external forces should be determined.

Generally, the following forces act when a ship crosses the channel bend:

- the forces of ship inertia (when stopping the ship or giving it the required speed);
- the forces of direct action of the hydrodynamic current;
- hydrodynamic “wing” forces (when the vessel is moving upstream, or is standing in the current);
- aerodynamic forces;
- forces resulting from the shallow water effect, etc.

The main parameters and variables used in the developed model are presented in Table 1.

Ship trajectory coordinates ($X_{0i},Y_{0i}$) during the crossing of channel bends can be calculated using Equations (1) and (2) [66]:

(1) $$X_{0i}=\int_{}v_{i}\cdot \cos(\int (\omega_{i}dt-\beta_{i}))dt+\int v_{cr}dt\cdot \cos q_{cr}+\int v_{d}dt\cos q_{a}$$

(2) $$Y_{0i}=\int v_{i}\cdot \sin(\int (\omega_{i}dt-\beta_{i}))dt+\int v_{cr}dt\cdot \sin q_{cr}+\int v_{d}dt\cdot \sin q_{a}$$

where $v_{i}$—ship’s speed; $\omega_{i}$—turning velocity; $\beta_{i}$—drift angle; $v_{cr}$—current velocity; $q_{cr}$—current course angle during start of the maneuvre; $v_{d}$—ship’s drift speed; $q_{a}$ wind course angle during start of the maneuverer. 

For the calculation of ship kinematic motion parameters, ship speed, turning speed and ship drift angle could be used. Forces and moments created by wind, current, waves, and shallow water effects acting on the ship were compensated by generating ship propulsion system forces and moments. Thus, the calculation of the forces and moments can be performed using the following Equations (3)–(5) [67]:

(3) $$X_{in}+X_{k}+X_{\beta }+X_{p}+X_{N}+X_{a}+X_{c}+X_{b}+X_{sh}+X_{T}+\ldots =0$$

(4) $$Y_{in}+Y_{k}+Y_{\beta }+Y_{p}+Y_{N}+Y_{a}+Y_{c}+Y_{b}+Y_{sh}+Y_{T}+\ldots =0$$

(5) $$M_{in}+M_{k}+M_{\beta }+M_{p}+M_{N}+M_{a}+M_{c}+M_{b}+M_{sh}+M_{T}+\ldots =0$$

where $X_{in},Y_{in},M_{in}$—inertia forces and the moment; $X_{k},Y_{k},M_{k}$—forces and moment created by the ship’s hull (may be calculated using the methodology presented in [66,67,68,69]); $X_{\beta },Y_{\beta },M_{\beta }$—the “wing”- related forces and the moment acting on ship’s hull (may be calculated using the methodology shown in [67,69]); $X_{p},Y_{p},M_{p}$—forces and the moment created by the ship’s rudder or other steering equipment (may be calculated using the methodology indicated in [67]); $X_{N},Y_{N},M_{N}$—forces and the moment created by thrusters; $X_{a},Y_{a},M_{a}$—aerodynamic forces and the moment (may be calculated using the methodology stated in [66,67,68,69]); $X_{c},X_{c},M_{c}$—forces and the moment created by the current (for calculations the methodology shown in [67,68,69] may be used); $X_{b},Y_{b},M_{b}$—forces and the moment created by waves (may be calculated using the methodology applied in [68,69]); $X_{sh},Y_{sh},M_{sh}$—forces and the moment created by shallow water effects (may be calculated by applying the methodology presented in [67]); $X_{T},Y_{T},M_{T}$—forces and the moment created by the ship’s propeller (propellers) (for calculations, the methodology indicated in [67,69] may be applied).

Additional forces and moments could be created by tugs, anchors or mooring ropes, or other factors, depending on the conditions of the ship’s movement through the channel bend. For the calculation of specified forces and moments, accurate measurements of internal and external factors’ influences and effects using the capabilities of available precise sensor systems are critically important.

While planning the ship’s trajectory during crossing the channel’s bends, very short time periods (no longer than 5–10 min) could be considered. It is assumed that during these periods, the current and ship’s drift could be taken as constant values. These parameters may be calculated by applying Equations (6) and (7):

(6) $$X_{0i}=\int_{}v_{i}\cdot \cos(\int (\omega_{i}dt-\beta_{i}))dt+v_{cr}\cdot t_{\psi }\cdot \cos q_{cr}+v_{d}{}_{}\cdot t_{\psi }\cdot \cos q_{a},$$

(7) $$Y_{0i}=\int v_{i}\cdot \sin(\int (\omega_{i}dt-\beta_{i}))dt+v_{cr}\cdot t_{\psi }\cdot \sin q_{cr}+v_{d}\cdot t_{\psi }\cdot \sin q_{a},$$

where $t_{\psi }$—ship’s turning (maneuvering) within channel bend time.

Currents that move a ship can be taken as constant on short sailing distances during the vessel’s crossing the channel bend. Moreover, the ship’s drift can be calculated considering the shallow water effect. Analyzing this effect, the ship’s drift velocity can be calculated using Equations (8) and (9):

(8) $$v_{dX}=\frac{v_{a}}{\sqrt{k_{R11}^{2}+k_{R22}^{2}}}\sqrt{\frac{C_{a}\cdot \rho_{1}\cdot \sqrt{S_{x}^{2}+S_{y}^{2}}\cdot \cos q_{a}}{C\cdot \rho \cdot \sqrt{F_{d}^{2}+W^{2}}}}$$

(9) $$v_{dY}=\frac{v_{a}}{\sqrt{k_{R11}^{2}+k_{R22}^{2}}}\sqrt{\frac{C_{a}\cdot \rho_{1}\cdot \sqrt{S_{x}^{2}+S_{y}^{2}}\cdot \sin q_{a}}{C\cdot \rho \cdot \sqrt{F_{d}^{2}+W^{2}}}}$$

where $v_{a}$—wind velocity; $k_{R11}$—the ship’s coefficient of resistance when the ship is moving straight taking into account clearance between ship’s hull and channel bottom or ratio *T/H* (ship’s draft and depths) (could be calculated using methodology shown in [67]); $k_{R22}$—the coefficient of resistance of a ship moving sideways, taking into account ratio *T/H* (could be calculated using methodology presented in [67]); $C_{a}$—aerodynamic coefficient, which in average is about 1.07 (specific data could be taken from aerodynamic tube testing); $\rho_{1}$—air density, for the calculations the value 1.25 kg/m^3^ may be taken; $S_{x}$—ship’s air projection on a diametric square; $S_{y}$—ship air projection on a middle square; C—hydrodynamic coefficient, could be taken as 1.5; ρ—water density; $F_{d}$—projection of the underwater part of the vessel into the diametrical plane; W—projection of the underwater part of the vessel into the middle plane.

In planning the process of ships crossing channel bends, it is also necessary to accurately calculate the additional forces and moments required to compensate for external forces [65]. Therefore, while conducting the calculations, it is very important to take into consideration that the trajectory of the ship crossing channel bends depends on the ship parameters, though the forces impacting the ship, created by wind and current, should be equivalent.

In the case where the ship’s maneuvering time of the crossing navigational channel bend is known, it is possible to calculate the distance crossed by the ship during maneuvers or the maximum wind speed that can move the ship during the crossing of the channel bend for a set distance. In this case, the ship’s movement distance, as a result of the acting current, wind, and shallow water effect, can be described by using Equations (10) and (11):

(10) $$X_{im}=t_{mX}(v_{cr}\cdot \cos q_{cr}+\frac{v_{a}}{\sqrt{k_{R11}^{2}+k_{R22}^{2}}}\sqrt{\frac{C_{a}\cdot \rho_{1}\cdot \sqrt{S_{x}^{2}+S_{y}^{2}}\cdot \cos q_{a}}{C\cdot \rho \cdot \sqrt{F_{d}^{2}+W^{2}}}})$$

(11) $$Y_{im}=t_{mY}(v_{cr}\cdot \sin q_{cr}+\frac{v_{a}}{\sqrt{k_{R11}^{2}+k_{R22}^{2}}}\sqrt{\frac{C_{a}\cdot \rho_{1}\cdot \sqrt{S_{x}^{2}+S_{y}^{2}}\cdot \sin q_{a}}{C\cdot \rho \cdot \sqrt{F_{d}^{2}+W^{2}}}})$$

where $t_{m}$—time for crossing channel bend by ship.

If the vessels’ movement distance in X or Y direction (the distance that the ship can sail during crossing channel bend) is known and current, wind, and shallow water parameters are determined, the vessels’ maneuvering time could be calculated by applying Equations (12) and (13):

(12) $$t_{mX}=\frac{X_{iX}}{v_{cr}\cdot \cos q_{cr}+\frac{v_{a}}{\sqrt{k_{R11}^{2}+k_{R22}^{2}}}\sqrt{\frac{C_{a}\cdot \rho_{1}\cdot \sqrt{S_{x}^{2}+S_{y}^{2}}\cdot \cos q_{a}}{C\cdot \rho \cdot \sqrt{F_{d}^{2}+W^{2}}}}}$$

(13) $$t_{mY}=\frac{Y_{iY}}{v_{cr}\cdot \sin q_{cr}+\frac{v_{a}}{\sqrt{k_{R11}^{2}+k_{R22}^{2}}}\sqrt{\frac{C_{a}\cdot \rho_{1}\cdot \sqrt{S_{x}^{2}+S_{y}^{2}}\cdot \sin q_{a}}{C\cdot \rho \cdot \sqrt{F_{d}^{2}+W^{2}}}}}$$

where $t_{mX}$—the ship’s maneuvering time during moving in the X direction; $t_{mY}$—the ship’s maneuvering time during moving in Y direction; $X_{iX}$—the ship’s maneuvering distance in X direction; $Y_{iY}$—the ship’s maneuvering distance in Y direction.

In the case of a ship is crossing a limited area, it is important to calculate, in advance, the wind velocity limitations, which means conditions under which the ship’s crossing channel bend could be safe. The limitation of wind velocity depends on the ships’ sailing direction and could be calculated as follows (Equations (14) and (15)):

(14) $$v_{a\lim X}=\frac{X_{mX}-t_{mX}\cdot v_{cr}\cdot \cos q_{cr}}{t_{mX}\cdot \sqrt{k_{R11}^{2}+k_{R22}^{2}}\sqrt{\frac{C_{a}\cdot \rho_{1}\sqrt{S_{x}^{2}+S_{y}^{2}}\cdot \cos q_{a}}{C\cdot \rho \cdot \sqrt{F_{d}^{2}+W^{2}}}}}$$

(15) $$v_{a\lim Y}=\frac{Y_{mY}-t_{mY}\cdot v_{cr}\cdot \sin q_{cr}}{t_{mY}\cdot \sqrt{k_{R11}^{2}+k_{R22}^{2}}\sqrt{\frac{C_{a}\cdot \rho_{1}\cdot \sqrt{S_{x}^{2}+S_{y}^{2}}\cdot \sin q_{a}}{C\cdot \rho \cdot \sqrt{F_{d}^{2}+W^{2}}}}}$$

where $v_{a\lim X}$—wind speed limitation that depends on the available distance $X_{mX}$ in maneuvering area and wind course angle; $v_{a\lim Y}$—wind speed limitation that depends on the available distance $Y_{mY}$ in maneuvering area and wind course angle $q_{a}$.

In complicated sailing conditions, very high accuracy of the ship’s position determination is critical. For the vessels’ position observation, RTK or laser systems can be used [1,9,18,45,52]. Applying the RTK system, the ship’s position determination accuracy may be achieved up to 5–10 cm; in this case, the sensors (reference station) are located within short distances (up to 1 mile). Updating the interval of the ship’s position provided by the RTK system is between 0.5–2.0 s; moreover, the ship’s sailing path and position could be predicted in 1, 3, 6, or 12 min, which is enough for monitoring ships’ sailing trajectory in complicated areas. In turn, laser systems ensure the determination of the ships’ position with an accuracy of up to 3–5 mm, however, the operating distance of these systems is very short, just up to 300–500 m. That is why these systems are used mainly in limited areas, inter alia, located close to the quay walls of terminals handling dangerous goods [52]. The ship’s position determination using laser systems [70,71] in complex channel bends by integrating wind, current, and other measurement results allows for minimizing discreteness and, at the same time, assess various effects influencing the vessel. This makes it possible to receive the exact position of the ship that may be seen on the electronic chart (after entering the geometric parameters of the ship into the sensor systems) and using the developed methodology to determine the expected trajectory of the ship and correct it in real-time. Such solutions may be used in the development of automated vessel’s management systems and autonomous ships.

The maximum distribution method [67,72] was used to evaluate the accuracy and reliability of the obtained calculations and experimental results.

## 4. Results

In order to investigate the limitations of the ship’s crossing channel bends within specific areas, the external forces caused by wind, current, waves, and shallow water effects during ship navigation were considered. Such conditions are typical for the ships crossing channel bends in areas located close to seaports. The strong influence of mentioned external forces is observed in archipelagos and fiords areas, such as the Stockholm archipelagos, in some locations near west shore of Finland, in Norway fjords, and other places.

**Figure 8:**
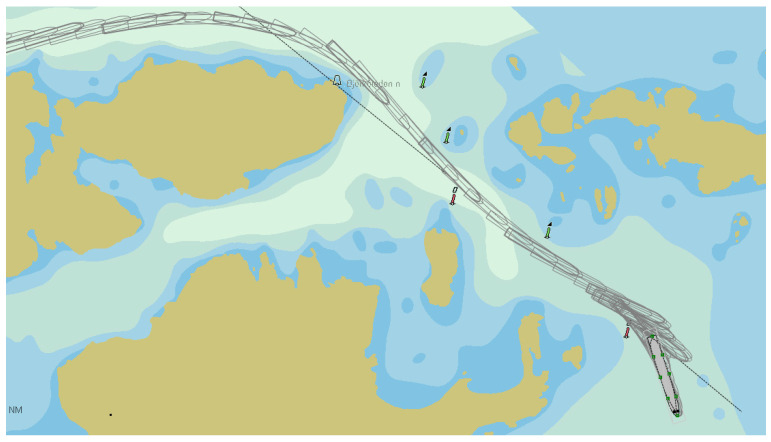
The navigational channel near Korsakobben Island and ship’s trajectory (Stockholm archipelago), where: buoys on chart are marked in black [73].

Experimental studies were carried out for two locations:

- the navigational channel near Korsakobben Island (Stockholm archipelago) for PANAMAX container vessel (Figure 8);
- the approach channel to Klaipeda port for the SUEZMAX tanker.

For the experimental testing of the simulator “SimFlex Navigator” [73] was used, as well as the real ships’ movement was tested.

The parameters of examined ships are shown in Table 2.

It was assumed that the PANAMAX container vessel with the container capacity of 4200 TEU moved within the navigational channel under the following conditions: north direction wind—10 m/s, waves—0.5 m, current—0.3 knots.

**Figure 9:**
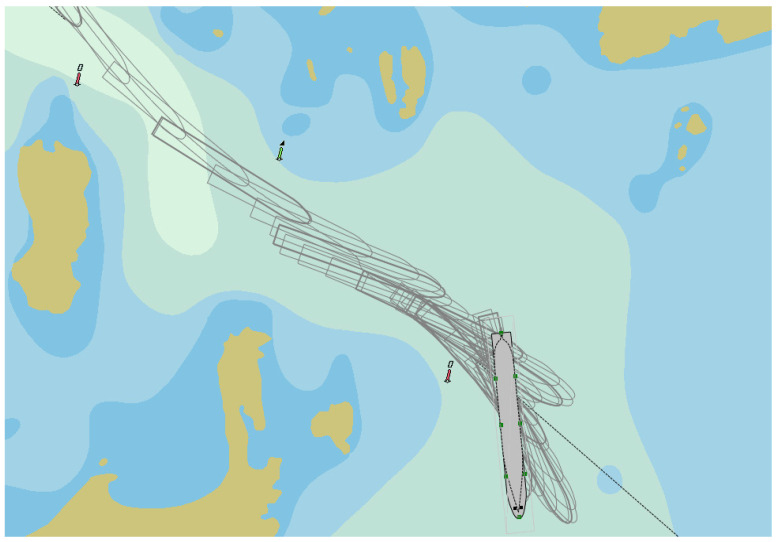
**A** PANAMAX-type container vessel crossing the navigational channel near Korsakobben Island (departure from Stockholm archipelago), where: buoys on chart are marked in black (own elaboration based on [73]).

**Figure 10:**
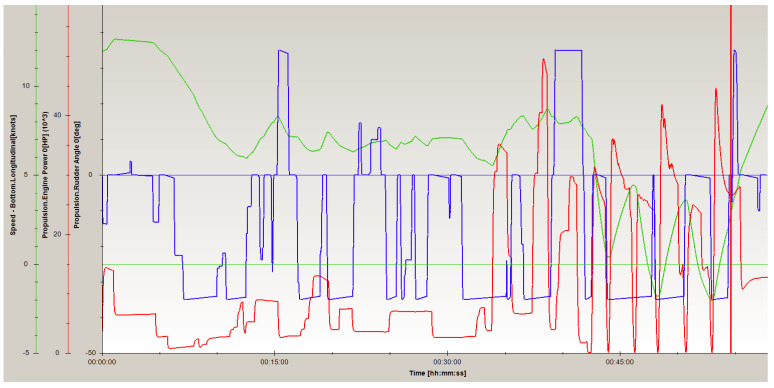
Sailing parameters of PANAMAX type container vessel crossing navigational channel near Korsakobben Island (departure from Stockholm archipelago), where: speed (green line); rudder angle (blue line); engine power (red line) (own elaboration based on [73]) (remark: −5; −50 in the figure read as −5; −50).

During crossing the navigational channel by PANAMAX type container vessel (Figure 9) near Korsakobben Island (departure from Stockholm archipelago), five ship‘s engine reverses were done. It was necessary because the distance between shallow places was about 300 m (the ship‘s length between perpendiculars was 284.7 m). The ship’s speed during maneuvers was not more than 2.5–2.8 knots. Depths in the maneuvering area was between 12.0–13.5 m and the ship‘s draft—10.5 m, therefore, a shallow water effect was observed that influenced the need to decrease the vessel’s drift speed more than two times. Maximum ship drift during maneuvers reached just up to 0.1–0.2 knots. The ship’s maneuvering time was about 11 min, and during this time, the ship’s drift distance was between 30–55 m. After the ship turned about 60 degrees, the drift speed decreased up to 0.03–0.05 knots. Maneuverer for crossing the channel’s bend lasted between 37 and 48 min. Engine power during maneuvers (engine reverses) was increased up to 60% from nominal engine power, which means the engine power reserves were made to assure the safety of performed operations. The vessel’s sailing parameters are presented in Figure 10.

**Figure 11:**
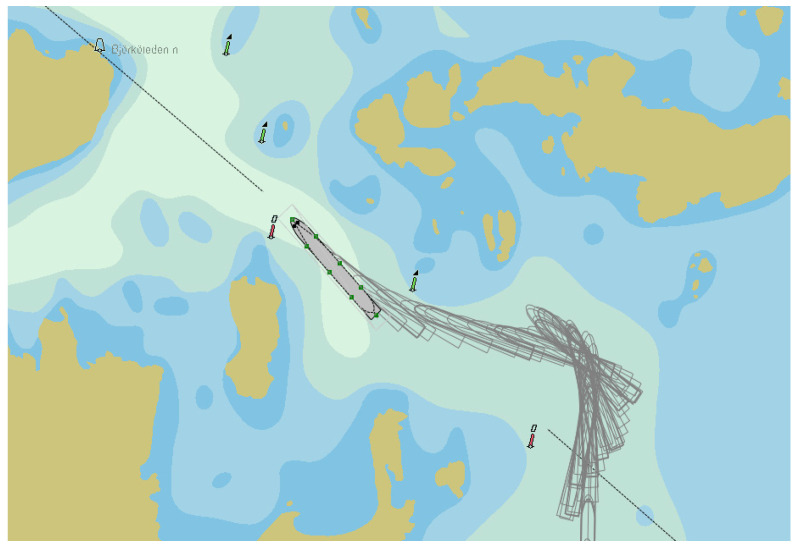
**A** PANAMAX-type container vessel crossing the navigational channel near Korsakobben Island (entry to Stockholm archipelago), where: buoys on chart are marked in black (own elaboration based on [73]).

**Figure 12:**
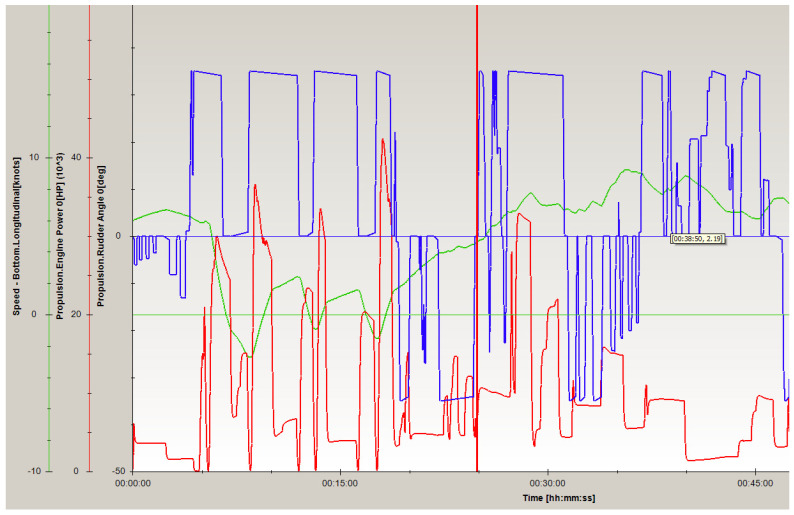
Sailing parameters of a PANAMAX-type container vessel crossing navigational channel near Korsakobben Island (entry to Stockholm archipelago from Baltic Sea): speed (green line); rudder angle (blue line); engine power (red line) (own elaboration based on [73]) (remark: −10; −50 in the figure read as −10; −50).

During a PANAMAX-type container vessel crossing a navigational channel bend in opposite direction (during entry to Stockholm archipelago) near Korsakobben Island (Figure 11). It was necessary to make a more than 90 degrees turn in similar conditions as in the case of departure (wind and current parameters were the same). During that process, six ship’s engine reverses were done (Figure 12). The vessel’s engine maneuvers during crossing the bend lasted from 6 up to 17 min.

It should be mentioned that the ship’s drift speed was 0.2 knots during the first stage of the container vessel’s maneuvers (minutes 6–8 from the start of the ships’ movement) and increased up to 0.3 knots, when the wind course angle reached 50 degrees (minutes 14–17 from the start of the ships’ movement). Shallow water affected the ship and influenced the decrease of the ship’s drift speed about 2–3 times.

Based on the achieved data analysis, it could be recommended that in order to obtain the high accuracy of the ship’s position, it is expedient to install RTK sensors (reference station) on the Korsakobben Island, that could determine the ship’s position with the accuracy of about 5–10 cm throughout this area and provide this information to vessel’s captain or pilot. This information may be very helpful to plan the ship’s maneuvers in real-time.

Another ship analyzed within the conducted research was a SUEZMAX tanker that sailed to Klaipeda port. The vessel’s movement within the navigational channel bend during entering the port was fixed by AIS and RTK system with an accuracy of 3–5 cm. Moreover, the data from the ship’s course and speed registration system was used to analyze the vessel’s trajectory. During the SUEZMAX tanker entering the port, the following sailing conditions took place: southwest wind—12 m/s, current out of port—0.5 knots, depth in channel—14.5 m.

**Figure 13:**
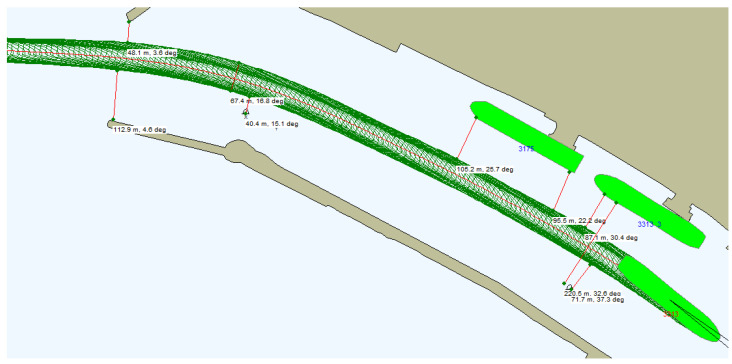
**A** SUEZMAX tanker’s sailing trajectory while crossing channel bend at the entrance of Klaipeda port (own elaboration based on [73]).

While the SUEZMAX tanker crossed the channel bend at the entrance to Klaipeda port, the ship’s engine reverses were not required, but the use of a maximal rudder angle was needed. Sometimes, tug assistance on the tanker’s stern should be used to increase the accuracy of the ship’s turning. It should be noted that during the ship’s entry to the port (Figure 13), the risks occur when sailing very close to the ship’s moored near oil terminal quay walls. This underlines the need to prepare maneuvering reverses very accurately, before crossing the navigational channel bend, and implementing them appropriately.

Analyzing the achieved results, it should be noted that, for example, the PANAMAX container vessel is able to cross the specified channel bend in 11 min (Figure 10 and Figure 12). During that time ship will be moved on the possible distance under the influence of current and the ship’s drift. Moreover, the shallow water effect may appear and decrease the ship’s drift speed. These parameters for the the PANAMAX container vessel during crossing the navigational channel near Korsakobben Island are presented in Table 3. The $v_{dX}/v_{dY}$ values differ depending on wind velocity ($v_{a}$) and wind course angle ($q_{a}$).

Applying the methodology presented in Section 3, using experimental results attained for the PANAMAX container vessel (Figure 10 and Figure 12) and considering the impact of the determined hydro-meteorological and hydrological conditions, the requested minimum maneuvering area ($X_{im}$ and $Y_{im}$) was calculated. The following assumptions were taken for computations: maneuvering time—660 s, current velocity—0.3 m/s, current course angle—30 degrees (in the start position of maneuver), wind course angle—70 degrees (in the start position of maneuver). 

The calculation results for the container vessel crossing the navigational channel bend near Korsakobben Island (departure and arrival from Stockholm archipelago), presenting the requested minimum maneuvering area and possible maneuvering time, are presented in Table 4 and Table 5.

In order to calculate wind velocity limitation while crossing the navigational channel bend, the SUEZMAX tanker entering the Klaipeda port was analyzed. The following parameters were considered for computations: depth in channel—14.5 m, ship’s air projection on diametric square ($S_{x}$)—4200 m^2^, ship air projection on middle square ($S_{y}$)—1300 m^2^, projection of the underwater part of the vessel into the diametrical plane ($F_{d}$)—3650 m^2^, projection of the underwater part of the vessel into the middle plane (W)—640 m^2^, current—0.5 m/s, current course angle—20 degrees (in maneuver start position), wind course angle—70 degrees (in maneuver start position). The results of wind velocity limitation calculations are presented in Table 6.

**Figure 14:**
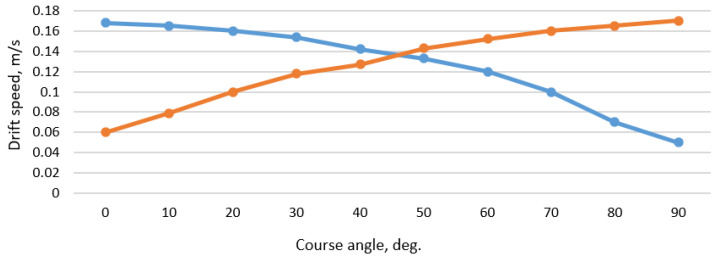
The SUEZMAX tanker drift speed ($v_{dX}$—blue line; $v_{dY}$—red line) during departure from Klaipeda port considering wind velocity 12 m/s and different wind course angles.

In the case of the maneuvering distances decrease up to $X_{mX}$ = 250 m and $Y_{mY}$ = 50 m, e.g., due to limited channel parameters on the way of ship’s departure from port, and changes in the wind direction take place, the SUEZMAX tanker’s drift speeds considering the permitted maximum wind velocity and course angle may be calculated. The results of these calculations are presented in Figure 14.

Wind velocity limitations depend on the wind direction (course angle). Wind velocity should be carefully registered and analyzed because, in many ports, wind velocity limitations are approved. However, these limitations usually do not consider wind direction. In such situations, an increased risk of accidents takes place, or ports apply a bigger safety margin and at the same time reduce ports efficiency. According to Klaipeda port regulations for ships, such as the SUEZMAX tanker entering the port, where the wind limitation is 12 m/s regardless of wind direction.

**Figure 15:**
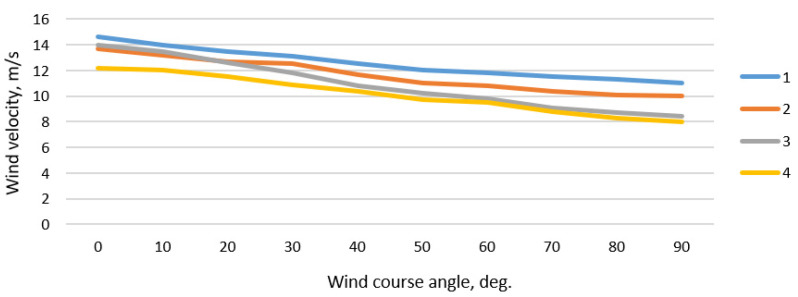
Limitations of wind velocity depending on wind course angle during ships departure from Klaipeda port, where: 1—LNG standard tanker; 2—SUEZMAX loaded tanker; 3—G class container vessel; 4—SUEZMAX tanker in ballast.

Based on the presented methodology, wind velocity limitations depending on wind direction for the SUEZMAX tanker and other selected types of ships during crossing navigational channel bend near Klaipeda port were calculated (Figure 15). These values may be used to better plan ships’ maneuvers in specific conditions. Moreover, achieved results may contribute and assist in preparing the navigational safety regulations in ports or other limited navigational areas.

Based on the presented case studies analysis, it could be stated that application of the presented methodology may allow to increase ports or channels accessibility without reduction of their navigational safety, especially when the parameters of external factors affecting the ships can be accurately determined with the help of sensors (measurement) systems.

## 5. Discussions and Conclusions

Complicated navigational conditions within the sharp and space-consuming bends of navigational channels take place in many ports’ entrances, fjords, and other areas. Possessing of an advanced theoretical basis and knowledge related to the ships’ movement in complicated conditions is needed for the channels’ designers, as well as ships’ captains and pilots, to prepare and perform safe ships maneuvers and decrease the risks of navigational accidents occurring.

The article presents the methodology that may be used for planning the process of channel bend crossing by different types of ships in specific conditions. Theoretical methods for the ships’ sailing parameter calculations and trajectories evaluation were presented and tested using simulators and data achieved from real ships moving in limited sailing conditions. The possibilities of calculating in advance the trajectory used by ships to cross the channel bends make it possible to increase safety levels and traffic efficiency in complicated and specific conditions of ships’ navigation. The presented approach for wind speed limitation evaluation can be used in practice during the preparation of ships’ maneuvers for crossing navigational channel bends.

The advantages of the proposed method in comparison with the existing ones are as follows:

- the possibility to assess the external boundary conditions with high accuracy depending on the ship’s controllability capabilities and channel parameters;
- with the use of data received from sensors and appropriate measurement systems, it is possible to calculate in advance the ship’s sailing trajectory with high accuracy, as well as possible maximum deviations from the planned trajectory;
- in case of deviations from the planned trajectory (with minimal discretion), it is possible to adjust the parameters of the ship’s movement without sailing outside the intended traffic lane, using the reversals of the ship’s main engine;
- implementation of measurement results achieved from sensors systems in the calculations of the sailing trajectory allows to minimize the discreteness of the trajectory estimation and, at the same time, the ship’s deviation from the planned trajectory,
- the developed methodology may be applied in advanced automated ship management systems and autonomous ships.

The developed mathematical model allows to calculate a ships’ trajectory, considering the dynamic behavior of ships in limited areas. In practice, it may be difficult to determine the exact coordinates of ships crossing the channel bends. Therefore, sensor systems and technologies (e.g., RTK) may be used to set the ship’s position and support the implementation of the presented approach.

Moreover, the presented methodology may form the basis for the elaboration and implementation of specific software that could be installed onboard the ships. This software may use data provided manually by the ships’ masters and be integrated with other systems that automatically transmit measurement results received from sensor systems. Such systems may deliver real-time data about currents, winds, shallow water effects, etc. Integration of data collected from RTK systems and data obtained by calculations based on the presented methodology, using real-time measurement results on external forces influencing the ship, gives the possibility to plan ships’ trajectories more accurately, especially in special navigational conditions.

It should be noted that the methodology was verified on selected examples (for instance, ship types, navigational channel bends) and considered the influence of chosen external factors according to set assumptions. Therefore, the results are limited to the selected areas that were investigated (crossing the navigational channel near Korsakobben Island and approaching to Klaipeda port). It is planned to verify methodology on the examples of other areas to examine the accuracy of trajectory planning in other limited areas for other types of ships. Nevertheless, its application is universal, and it may be used to analyze different cases.

The research made it possible to investigate more deeply how the vessels’ dynamics and external factors influence these ships. It should be highlighted that selected external factors influencing the ships’ maneuvering were distinguished. Therefore, the expansion of the range of factors and the potential possibility to consider them in a mathematical model may be considered in future research.

Comparing the results achieved on the simulators and on real ships, it could be stated that the presented methodology implementation allowed for accomplishing a good correlation between theoretical calculations and experimental results. That fact provides to the conclusion that shown approach may be used for solving practical tasks.

The research results may be useful for ships’ captains and officers, as well as port pilots, who perform the operations of ships entering approaches to ports and channel bends. Application of presented theoretical methods may be made before ships start the maneuvering in specific conditions, while planning and preparing for crossing the specific limited areas. Moreover, the approach may be used during the operations performance, that will give the possibility to provide corrections to the earlier planned trajectory. Furthermore, this methodology may also be utilized in inland navigation when ships are moving in narrow channels that have limited sailing conditions.

The application of methodology may allow big vessels to cross the channel bends and complicated areas, limit cost-consuming investments in dredging the channels and approaches to ports, and at the same time keep the safety level of ships navigation (not lower than with investments implementation). Furthermore, results of simulations conducted using the presented methodology may form the basis for changes in rules applied in ships movement in limited areas, for example in seaports.

Our future research will focus on a more detailed investigation of ships’ movement within specific navigational areas using new sensors systems. Simulations of ship maneuvers will be done, and the impact of decisions taken by ship’s crew on the level of ship safety will be investigated. Moreover, a detailed analysis of how weather conditions influence on the ships movement in channel bends will be carried out.

## Figures and Tables

**Table 1:** Main parameters and variables used in the model.

Parameters	Parameters Explanation
$X_{0i},Y_{0i}$	Ship’s sailing trajectory coordinates.
$v_{dX},v_{dY}$	Ship’s drift velocity on *X* and *Y* directions.
$X_{im},Y_{im}$	Ship’s movement distance on *X* and *Y* directions as a result of the acting current, wind, and shallow water effect.
$t_{mX}^{},t_{mY}$	Ships’ maneuvering time on *X* and *Y* directions.
$v_{a\lim X},v_{a\lim Y}$	Limitation of wind velocity depending on ships’ sailing on *X* and *Y* directions.

**Table 2:** Selected parameters of analyzed ships [18,23,67].

Parameter	PANAMAX Container Vessel	SUEZMAX Tanker
Length, m	295	274
Length between perpendiculars, m	284.7	261
Width, m	32.2	49
Average draft, m	10.5	13
Full speed, knots	25.3	15
Displacement, t	61,590	127,000

**Table 3:** The PANAMAX container vessel drifts speed depending on wind velocity and course angle ($v_{dX}/v_{dY}$ ) during crossing the navigational channel near Korsakobben Island (departure from Stockholm archipelago).

$v_{a},\;m/s$	$q_{a}$						
	0°	30°	60°	90°	120°	150°	180°
5	0.07/0	0.06/0.05	0.05/0.07	0/0.07	−0.51/0.07	−0.07/0.05	−0.07/0
10	0.15/0	0.14/0.10	0.10/0.14	0/0.15	−0.10/0.14	−0.14/0.10	−0.15/0
15	0.22/0	0.20/0.15	0.15/0.20	0/0.22	−0.15/0.20	−0.20/0.15	−0.22/0
20	0.29/0	0.27/0.21	0.21/0.27	0/0.29	−0.21/0.27	−0.27/0.21	−0.29/0

**Table 4:** The requested minimum maneuvering area within channel bend depending on wind velocity.

Wind Velocity, m/s	5	10	15	20
$X_{im}$, m	186	202	218	234
$Y_{im}$, m	133	176	215	262

**Table 5:** Possible maneuvering time within channel bend depending on wind velocity ($X_{im}$ = 250 m; $Y_{im}$ = 180 m).

Wind Velocity, m/s	5	10	15	20
$t_{mX}$, s/min	881/14, 7	814/13, 6	756/12, 6	714/11, 9
$t_{mY}$, s/min	837/14, 0	643/10, 7	522/8, 7	439/7, 3

**Table 6:** Wind velocity limitation in case of $X_{mX}$ —700 m and $Y_{mY}$ —350 m.

$t_{m},\;s$	200	220	240	260
$v_{a\lim X}$, m/s	17.4	15.7	14.0	12.8
$v_{a\lim Y}$, m/s	10.3	8.7	7.9	7.2

## Data Availability

Not applicable.
